# Impact of the HLA-DRB1 shared epitope on responses to treatment with tofacitinib or abatacept in patients with rheumatoid arthritis

**DOI:** 10.1186/s13075-021-02612-w

**Published:** 2021-08-31

**Authors:** Wataru Hirose, Masayoshi Harigai, Koichi Amano, Toshihiko Hidaka, Kenji Itoh, Kazutoshi Aoki, Masahiro Nakashima, Hayato Nagasawa, Yukiko Komano, Toshihiro Nanki, for the TOF-ABT Study Group, Yuji Akiyama, Souichirou Ando, Yayoi Hashiba, Motohide Kaneko, Mitsuhiro Kawagoe, Tsuneo Kondo, Kazuyoshi Kubo, Ikuko Masuda, Mitsuyo Matsumoto, Yusuke Okada, Akiko Shibata, Kimihiro Suzuki, Ko Takamatsu, Hirofumi Takei

**Affiliations:** 1 Hirose Clinic of Rheumatology, 2-14-7 Midori-chou, Tokorozawa, Saitama, 359-1111 Japan; 2 Division of Rheumatology, Department of Internal Medicine, Tokyo Women’s Medical University School of Medicine, Shinjuku-ku, Tokyo, Japan; 3 Department of Rheumatology and Clinical Immunology, Saitama Medical Center, Saitama Medical University, Kawagoe, Saitama, Japan; 4 Institute of Rheumatology, Zenjinkai Shimin no mori Hospital, Miyazaki city, Miyazaki, Japan; 5 Division of Rheumatology, Department of Internal Medicine, National Defense Medical College, Tokorozawa, Saitama, Japan; 6 Aoki Clinic of Rheumatology, Saitama city, Saitama, Japan; 7 Department of Immunology and Microbiology, National Defense Medical College, Tokorozawa, Saitama, Japan; 8 Nagasawa Clinic of Rheumatology, Kawagoe, Saitama, Japan; 9 Division of Rheumatology, Department of Internal Medicine, Jujo Takeda Rehabilitation Hospital, Minami-ku, Kyoto, Japan; 10 Division of Rheumatology, Department of Internal Medicine, Toho University School of Medicine, Ota-ku, Tokyo, Japan

**Keywords:** Rheumatoid arthritis, Shared epitope, Tofacitinib, Abatacept, Propensity score matching

## Abstract

**Objectives:**

The aim of this study was to compare the clinical effectiveness of tofacitinib and abatacept and clarify the impact of the HLA-DRB1 shared epitope (SE) on responses to these treatments in patients with rheumatoid arthritis (RA).

**Methods:**

After adjustments by propensity score matching, 70 out of 161 patients receiving tofacitinib and 70 out of 131 receiving abatacept were extracted. The clinical effectiveness of both drugs over 24 weeks and the impact of the copy numbers of SE on effectiveness outcomes were investigated.

**Results:**

The percentage of patients in remission in the 28-joint count disease activity score using the erythrocyte sedimentation rate (DAS28-ESR) did not significantly differ between patients receiving tofacitinib and abatacept at week 24 (32% vs 37%, *p* = 0.359). The mean change at week 4 in DAS28-ESR from baseline was significantly greater in patients receiving tofacitinib than in those receiving abatacept (− 1.516 vs − 0.827, *p* = 0.0003). The percentage of patients in remission at week 4 was 30% with tofacitinib and 15% with abatacept (*p* = 0.016). When patients were stratified by the copy numbers of SE alleles, differences in these numbers did not affect DAS28-ESR scores of patients receiving tofacitinib. However, among patients receiving abatacept, DAS28-ESR scores were significantly lower in patients carrying 2 copies of SE alleles than in those carrying 0 copies at each time point throughout the 24-week period. Furthermore, the percentage of patients in remission with DAS28-ESR at week 24 was not affected by the copy numbers of SE alleles in patients receiving tofacitinib (*p* = 0.947), whereas it significantly increased as the copy numbers became higher in patients receiving abatacept (*p* = 0.00309). Multivariable logistic regression analyses showed a correlation between the presence of SE and DAS28-ESR remission in patients receiving abatacept (OR = 25.881, 95% CI = 3.140–213.351, *p* = 0.0025), but not in those receiving tofacitinib (OR = 1.473, 95% CI = 0.291–7.446, *p* = 0.639).

**Conclusions:**

Although the clinical effectiveness of tofacitinib and abatacept was similar at week 24, tofacitinib was superior to abatacept for changes from baseline in DAS28-ESR and the achievement of remission at week 4. SE positivity was associated with the achievement of DAS28-ESR remission by week 24 in patients receiving abatacept, but not in those receiving tofacitinib.

**Supplementary Information:**

The online version contains supplementary material available at 10.1186/s13075-021-02612-w.

## Background

Rheumatoid arthritis (RA) is a complex autoimmune disease that develops from the combined effects of genetic and environmental factors. Genetic factors make a significant contribution to the development of RA in population, accounting for approximately 60% of population susceptibility to the disease [1]. Among the susceptibility genes to RA, the strongest relationship was reported with the HLA region, particularly HLA-DRB1 alleles that share a similar amino acid sequence, called the shared epitope (SE) [2]. Although the SE hypothesis was initially proposed to explain genetic susceptibility to RA, subsequent investigations suggested that the primary role of SE may be in the development of more severe disease manifestations [3, 4]. The well-known SE-coding alleles include members of the HLA-DRB1 *04 allele group, *0101, *1402, and *1001 [5]. SE may modulate the severity of RA in affected patients [3, 4, 6]. Autoantibodies, such as the anti-citrullinated peptide antibody (ACPA), are more likely to occur in patients with RA who are positive for SE [7–9]. SE has also been linked to progressive joint damage [10]. A recent study revealed that the presence of Val and Leu at HLA-DRB1 position 11, other than SE, were associated with more radiographic progression [11]. Furthermore, the presence of the SE may affect responses to treatment [12, 13]. However, the role of SE alleles in therapeutic responses remains unclear.

The introduction of multiple biologic disease modifying antirheumatic drugs (bDMARDs) and targeted synthetic DMARDs (tsDMARDs) has significantly improved the treatment of RA. According to the European League Against Rheumatism (EULAR) 2019 update recommendations, if the treatment target is not achieved with the first conventional synthetic DMARD (csDMARD) strategy and poor prognostic factors are present, bDMARD or tsDMARD is recommended in the next step [14]. Among candidate drugs, tofacitinib is an oral Janus kinase (JAK) inhibitor that preferentially reduces signaling from type I and II cytokine receptors by inhibiting JAK3 and/or JAK1 [15, 16], while abatacept is a genetically engineered fusion protein that selectively inhibits T cell activation by binding to CD 80/86 and modulating their interaction with CD28 [17]. Both tofacitinib and abatacept have been shown to be similarly efficacious based on clinical outcomes in randomized controlled trials (RCTs) compared to adalimumab [18, 19]. In Japan, abatacept use comprised 17% of overall bDMARDs use in fiscal year 2017 and the proportion was higher especially in elderly populations [20], indicating that a comparative effectiveness study of abatacept with targeted therapies other than TNF inhibitors is necessary for clinical decision making. However, to date, there are no clinical studies that directly compare the efficacies of both drugs. Therefore, this study was undertaken as the first comparison of tofacitinib and abatacept for the treatment of RA. RCTs are regarded as a reliable means of obtaining evidence on the efficacy and safety of drugs. However, there are limitations to RCTs. The most important limitation is that the study participants are selected using inclusion and exclusion criteria. On the other hand, observational studies involve patients who are commonly encountered in daily clinical practice. But the study participants are subjected to selection bias due to uncontrolled differences between the treatment and control groups. Therefore, a precise comparison of the effectiveness of drugs is difficult in observational studies. In recent years, propensity score (PS) matching has been shown to reduce limitations, such as selection bias arising from observational studies, by adjusting for potential confounders and producing similar data to RCTs [21, 22].

In the present study, we used the PS matching method to compare clinical outcomes for 24 weeks between patients receiving tofacitinib and abatacept and attempted to clarify whether the copy numbers of SE alleles affect responses to treatment with tofacitinib or abatacept during 24 weeks in patients with RA.

## Methods

### Patients and study design

This was a multicenter, retrospective, longitudinal observational study conducted at 12 hospitals and clinics for rheumatology in Japan. We enrolled patients aged ≥ 20 years who fulfilled the 2010 American College of Rheumatology (ACR)/EULAR criteria [23] for RA and started treatment with tofacitinib or abatacept between January 2015 and September 2019. The prior use of bDMARDs or JAK inhibitors did not limit patient enrollment in the present study. Data in this study were collected prospectively from January 2018 as well as retrospectively for patients who had been treated with tofacitinib or abatacept until December 2017. The clinical effectiveness of the tofacitinib and abatacept treatments was evaluated over 24 weeks. An HLA-DRB1 allele analysis was performed at enrollment. Written informed consent was obtained according to the Declaration of Helsinki. This study design was initially approved by the Ethics Committee of Toho University School of Medicine in January 2018 (approved number, A17085) and then by each participating center or institution. This study was registered with the University Hospital Medical Information Network Clinical Trial Registry (UMIN000037418).

### Tofacitinib and abatacept treatments

Tofacitinib and abatacept were prescribed to patients with RA at the discretion of the treating physician. The dosage of tofacitinib was adjusted by renal function. Patients with an estimated glomerular filtration rate (eGFR) ≥ 60 ml/min/1.73m^2^ received 5 mg of tofacitinib orally twice daily, while those with eGFR< 60 ml/min/1.73m^2^ received 5 mg of tofacitinib orally once daily. Abatacept was administered as an intravenous infusion (500 mg for patients of < 60 kg, 750 mg for 60–100 kg and 1000 mg for > 100 kg) on weeks 0, 2, and 4, and then every 4 weeks thereafter. Alternatively, patients received 125 mg by a subcutaneous injection once weekly [24].

### Clinical effectiveness

Disease activity was assessed by the 28-joint count disease activity score using the erythrocyte sedimentation rate (DAS28-ESR) [25], the Simplified Disease Activity Index (SDAI) [26], and the Clinical Disease Activity Index (CDAI) [27] at baseline, 4, 12, and 24 weeks. The EULAR response was evaluated at 4, 12, and 24 weeks [28].

### HLA-DRB1 genotyping and autoantibody detection

The HLA-DRB1 allele was genotyped by the SeCore DRB1 Locus Exon 2 & 3 Sequencing kit (One Lambda) with the polymerase chain reaction-sequencing based typing method. HLA-DRB1 *01:01, *04:01, *04:04, *04:05, *04:10, *10:01, *14:02, and *14:06 were defined as SE [29]. ACPA was detected using a second-generation anti-CCP CLIA kit (Abbott Japan Laboratories, Tokyo, Japan). A cutoff value of 4.5 U/ml was used for anti-CCP antibody positivity.

### Statistical analysis

We compared the baseline characteristics of patients treated with tofacitinib and abatacept. To simultaneously control for potential confounders, we generated PS to predict the probability of a patient initiating tofacitinib by a multiple logistic regression model using the following key variables at baseline: age, sex, disease duration, body mass index, number of SE, biologic-naïve, tender joint count (TJC), swollen joint count (SJC), patient’s global assessment (PGA), physician’s global assessment (PGA), DAS28-ESR, the rheumatoid factor (RF) titer, ACPA titer, ESR, C-reactive protein (CRP), number of lymphocytes, hemoglobin, and Health Assessment Questionnaires Disability Index (HAQ-DI). We then performed 1:1 nearest neighbor matching using a caliper of 1 of the standard deviation of the logit of the PS scale for tofacitinib [30, 31]. After confirming the sufficient accuracy of this method (the area under the receiver operating characteristic curve was 0.86 when all samples before PS matching was used.), we matched 70 patients to each group. Differences between groups of normally distributed continuous data were examined using the Student’s *t* test. Differences between groups of non-normally distributed continuous data were tested for significance as follows: non-parametric Mann-Whitney *U* test with Bonferroni corrections to compare two groups and the Kruskal-Wallis test to compare three groups. Pearson’s *χ*^2^ test was used for categorized variables. The Kaplan-Meier method was used to assess retention rates and differences were analyzed by the Log-rank test. The effects of the number of SE copies on the proportion of patients who achieved a good EULAR response or DAS28-ESR remission at 24 weeks in each treatment group were assessed using the Cochran-Armitage test. The impact of SE on DAS28-ESR at week 24 was examined by a multivariable conditional logistic regression model, which was adjusted for age, sex, disease duration, biologic-naïve, the ACPA titer, HAQ-DI at baseline, and ΔDAS28, which indicates the magnitude of changes from baseline to week 4 in DAS28-ESR after PS matching. *P* values less than 0.05 were considered to be significant. The last observation carried forward method was used for patients who discontinued treatment before week 24 to include all patients in the analysis. All statistical analyses were performed with R version 3.6.1 (R Core Team, 2019, Vienna, Austria).

## Results

### Enrollment of study participants and baseline characteristics

One hundred and sixty-four patients treated with tofacitinib and 131 patients treated with abatacept were enrolled (see Supplementary Table S1). All patients provided written informed consent for the present study. To avoid treatment-selection bias, PS matching was performed, resulting in 70 matched pairs of patients treated with tofacitinib or abatacept. No significant differences were observed in the baseline characteristics of the two groups (Table 1). Unless otherwise stated, data after the PS matching was used for subsequent analyses.

**Table 1:** Baseline characteristics of patients after propensity score matching

Variables	Tofacitinib (*n* = 70)	Abatacept (*n* = 70)	*P*
Age, years	69.2 ± 10.1	68.6 ± 12.1	0.925
Female, *n*, %	59 (84.3)	58 (82.9)	1.000
Disease duration, years	14.8 ± 14.0	14.1 ± 13.0	0.678
Stage I/II/III/IV, %	20.0/10.0/32.9/37.1	25.7/12.9/32.9/28.6	0.694
Class 1/2/3/4, %	5.7/71.4/22.9/0.0	5.7/68.6/25.7/0.0	0.956
BMI, kg/m^2^	21.4 ± 3.3	21.8 ± 2.8	0.505
SE copy number 0/1/2, %	31.4/61.4/7.1	28.6/54.3/17.1	0.252
Current smoker, *n* (%)	4 (5.7)	5 (7.1)	1.000
Ever smoker, *n* (%)	14 (20.0)	21 (30.0)	0.241
No. of prior biologic use			
0 (biologic naïve )	31	37	0.398
1	13	14	0.830
2	14	7	0.098
≥ 3	12	12	1.00
MTX use, *n* (%)	41 (58.6)	40 (57.1)	1.000
MTX dose, mg/week	8.4 ± 2.4	8.0 ± 2.7	0.441
Oral corticosteroid use, *n* (%)	27 (38.6)	24 (34.3)	0.726
Oral corticosteroid dose, mg/day^a^	4.5 ± 2.8	5.5 ± 3.9	0.288
MMP-3, ng/mL	243.6 ± 242.9	153.4 ± 134.6	0.140
SJC, 0–28	4.4 ± 4.1	3.7 ± 3.7	0.507
TJC, 0–28	5.9 ± 5.0	5.4 ± 5.3	0.557
ESR, mm/h	40.1 ± 32.0	38.2 ± 30.2	0.882
CRP, mg/dL	1.54 ± 1.92	1.48 ± 2.19	0.742
RF, U/mL	217.7 ± 529.9	165.1 ± 375.8	0.790
ACPA, U/mL	245 ± 393.3	256.6 ± 293.8	0.323
GH, VAS 0–100 mm	53.1 ± 28.4	52.2 ± 24.2	0.777
EGA, VAS 0–100 mm	47.8 ± 20.4	45.2 ± 15.8	0.522
SDAI	21.9 ± 12.1	20.3 ± 11.4	0.283
CDAI	20.3 ± 11.2	19.8 ± 10.0	0.326
DAS28-ESR	4.8 ± 1.4	4.7 ± 1.3	0.508
HAQ-DI	1.12 ± 0.81	1.07 ± 0.76	0.689

^a^Prednisolone equivalents

Results are expressed as means ± SD unless otherwise stated

Comparisons of matched groups were performed using the Student’s *t* test for continuous variables and Pearson’s *χ*^2^ test for categorized variables. *ACPA* anticitrullinated peptide antibody, *BMI* body mass index, *CRP* C-reactive protein, *CDAI* Clinical Disease Activity Index, *DAS28-ESR* Disease Activity Score in 28 joints using the erythrocyte sedimentation rate, *EGA* evaluator global assessment of disease activity, *ESR* erythrocyte sedimentation rate, *GH* patient’s global assessment of general health, *HAQ-DI* Health Assessment Questionnaire Disability Index, *MMP-3*, matrix metalloproteinase 3, *MTX* methotrexate, *RF* rheumatoid factor, *SE* shared epitope, *SDAI* Simplified Disease Activity Index, *SJC* swollen joint count, *TJC* tender joint count

### Comparison of clinical efficacies between tofacitinib and abatacept

**Fig. 1:**
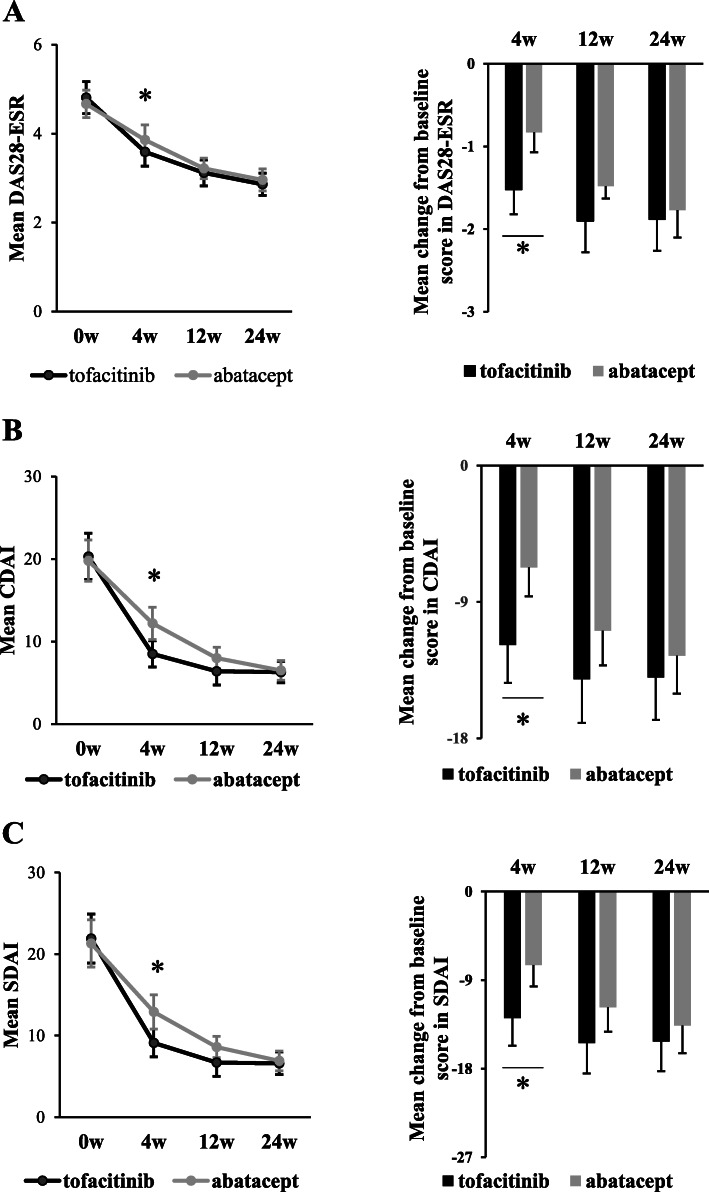
Disease activity scores in DAS28-ESR, CDAI, and SDAI. Mean disease activity scores and mean changes from baseline scores in DAS28-ESR (**A**), CDAI (**B**), and SDAI (**C**) recorded over 24 weeks are shown after the initiation of treatment with tofacitinib or abatacept. RA disease activity scores between the two treatment groups were compared at each time point. Error bars indicate 95% confidence intervals. *p < 0.05 by the Student’s *t* test. CDAI, Clinical Disease Activity Index; DAS28-ESR, Disease Activity Score in 28 joints using the erythrocyte sedimentation rate: SDAI, Simplified Disease Activity Index

**Fig. 2:**
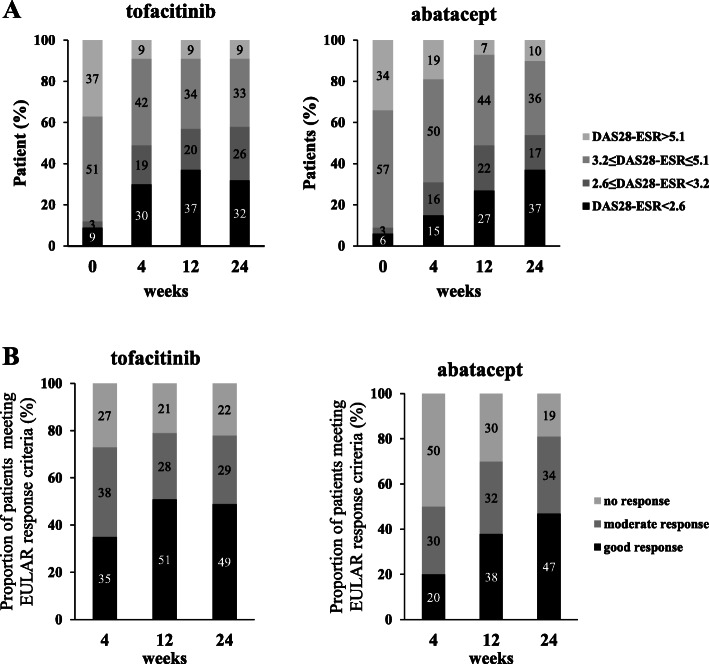
Time course of DAS28-ESR and EULAR responses. **A** Changes in disease activity categories according to DAS28-ESR are shown at baseline, 4, 12, and 24 weeks after the treatment with tofacitinib or abatacept. **B** The proportion of patients meeting the EULAR response criteria are shown 4, 12, and 24 weeks after the start of treatment with tofacitinib or abatacept. The percentage of patients who achieved good or moderate EULAR responses or DAS28-ESR remission were compared between the two treatment groups at each time point. DAS28-ESR, Disease Activity Score in 28 joints using the erythrocyte sedimentation rate; EULAR, European League Against Rheumatism

Retention rates are shown in Supplementary Figure S1. At week 24, 92.9% of patients in both groups were still receiving tofacitinib or abatacept. Retention rates were not significantly different between the two groups (*p* = 0.606 by Log-rank test for evaluating time to discontinuation). The disease activity scores of DAS28-ESR, CDAI, and SDAI recorded over 24 weeks are shown in Fig. 1. No significant differences were observed in improved DAS28-ESR, CDAI, or SDAI scores at week 24 between the two treatment groups. However, tofacitinib was superior to abatacept for the mean disease activity scores of DAS28-ESR (tofacitinib vs abatacept; 3.3 vs 3.9, *p* = 0.011), CDAI (tofacitinib vs abatacept; 8.5 vs 12.2, *p* = 0.0043), and SDAI (tofacitinib vs abatacept; 9.1 vs 12.9, *p* = 0.0037) at week 4 as well as for the mean changes from baseline at week 4 in DAS28-ESR (tofacitinib vs abatacept; − 1.52 vs − 0.83, *p* = 0.0003), CDAI (tofacitinib vs abatacept; − 11.86 vs − 6.73, *p* = 0.00087), and SDAI (tofacitinib vs abatacept; − 12.86 vs − 7.46, *p* = 0.0018) scores. Changes in disease activity categories according to DAS28-ESR are shown in Fig. 2 A. Tofacitinib was superior to abatacept for the percentage of patients in DAS28-ESR remission at week 4 (tofacitinib vs abatacept; 30% vs 15%, *p* = 0.016), while no significant difference was observed at week 24 (tofacitinib vs abatacept; 32% vs 37%, *p* = 0.359). Changes in disease activity categories according to CDAI and SDAI were also examined as shown in Supplementary Figure S2. Tofacitinib was superior to abatacept for the percentage of patients in remission at week 4 (tofacitinib vs abatacept; 20% vs 7%, *p* = 0.044) and week 12 (tofacitinib vs abatacept; 34% vs 17%, *p* = 0.016) in CDAI and at week 12 (tofacitinib vs abatacept; 34% vs 16%, *p* = 0.022) in SDAI, while no significant differences were observed in the percentage of patients in remission in CDAI and SDAI at week 24 between the two groups.

The time courses of EULAR responses are shown in Fig. 2 B. At week 24, the percentages of patients who achieved good or moderate EULAR responses in the tofacitinib and abatacept groups were 78 and 81%, respectively. No significant differences were observed between the two treatment groups (*p* = 0.72). However, at week 4, the percentage of good or moderate EULAR responses was significantly higher in the tofacitinib group than in the abatacept group (tofacitinib vs abatacept; 73% vs 50%, *p* = 0.0026). The proportion of patients with a good EULAR response reached a plateau at week 12 in the tofacitinib group, while it continuously increased for 24 weeks in the abatacept group.

### Influence of HLA-DRB1 SE on responses to treatment

**Fig. 3:**
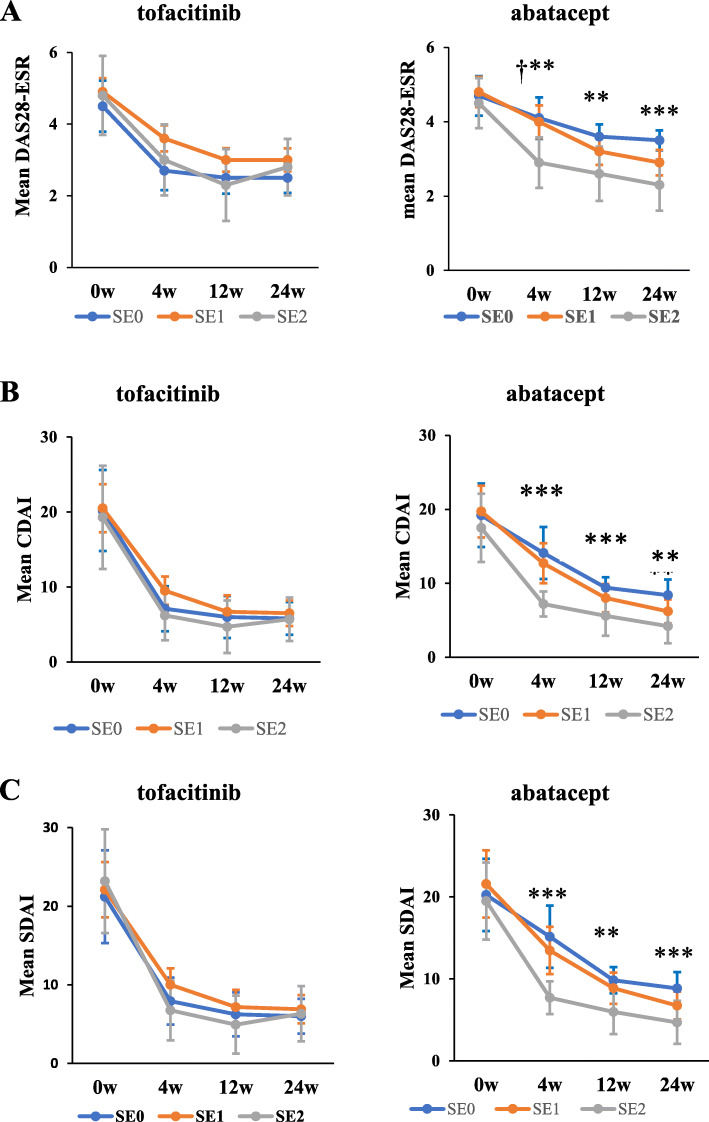
Copy numbers of SE alleles and RA disease activity scores. The mean values of scores in DAS28-ESR (**A**), CDAI (**B**), and SDAI (**C**) stratified according to the copy number of SE alleles are shown at baseline, 4, 12, and 24 weeks after the start of treatment with tofacitinib or abatacept. RA disease activity scores in DAS28-ESR, CDAI and SDAI were compared between 0 SE alleles and 1 SE allele, 0 SE alleles and 2 SE alleles, and 1 SE allele and 2 SE alleles at each time point in each treatment by the Mann-Whitney *U* test with Bonferroni corrections. Error bars indicate 95% confidence intervals. *P* values less than 0.0167 were considered to be significant. †*p* < 0.0167, 1 SE allele and 2 SE alleles were compared; **p < 0.0167, 0 SE alleles and 2 SE alleles were compared; ***p < 0.0033, 0 SE alleles and 2 SE alleles were compared. CDAI, Clinical Disease Activity Index; DAS28-ESR, Disease Activity Score in 28 joints using the erythrocyte sedimentation rate; SDAI, Simplified Disease Activity Index; RA, rheumatoid arthritis; SE 0, 0 copies of shared epitope alleles; SE 1, 1 copy of shared epitope alleles; SE 2, 2 copies of shared epitope alleles

The time courses of scores in DAS28-ESR, CDAI, and SDAI stratified according to the copy numbers of SE alleles are shown in Fig. 3. RA disease activity scores in each treatment group at each time point were compared between 0 SE alleles and 1 SE allele, 0 SE alleles and 2 SE alleles, and 1 SE allele and 2 SE alleles by the Mann-Whitney *U* test with Bonferroni corrections which adjust *p* values less than 0.0167 to be significant. In patients receiving tofacitinib, no significant differences were observed in RA disease activity scores regardless of the copy numbers of SE alleles and the composite measures for disease activities used in the analyses. In contrast, in patients receiving abatacept, significant differences were observed in RA disease activity scores that depended on the copy numbers of SE alleles. The following analyses showed significant differences in DAS28-ESR: 1 SE allele vs 2 SE alleles (4.0 vs 2.9, *p* = 0.0127) and 0 SE alleles vs 2 SE alleles (4.1 vs 2.9, *p* = 0.00986) at week 4, 0 SE alleles vs 2 SE alleles (3.6 vs 2.6, *p* = 0.0106) at week 12, and 0 SE alleles vs 2 SE alleles (3.5 vs 2.3, *p* = 0.000959) at week 24 (Fig. 3 A). The following significant differences were observed in CDAI: 0 SE alleles vs 2 SE alleles (14.1 vs 7.2, *p* = 0.00162) at week 4, 0 SE alleles vs 2 SE alleles (9.4 vs 5.5, *p* = 0.00167) at week 12, and 0 SE alleles vs 2 SE alleles (8.4 vs 4.2, *p* = 0.00456) at week 24 (Fig. 3 B). The following significant differences were observed in SDAI: 0 SE alleles vs 2 SE alleles (15.1 vs 7.7, *p* = 0.00240) at week 4, 0 SE alleles vs 2 SE alleles (9.8 vs 6.0, *p* = 0.0150) at week 12, and 0 SE alleles vs 2 SE alleles (8.8 vs 4.7, *p* = 0.00173) at week 24 (Fig. 3 C).

**Fig. 4:**
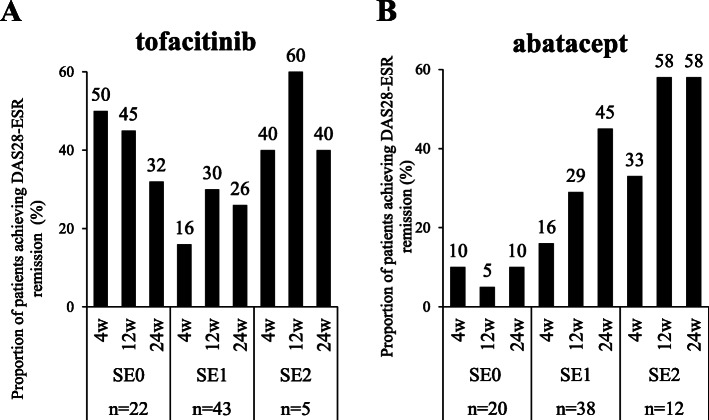
Copy numbers of SE alleles and remission in DAS28-ESR. The percentage of patients in DAS28-ESR remission stratified according to the copy numbers of SE alleles are shown 4, 12, and 24 weeks after the start of treatment with tofacitinib (**A**) or abatacept (**B**). The effects of the copy numbers of SE alleles on the percentage of patients achieving remission in DAS28-ESR at 24 weeks in each treatment group were assessed by the Cochran-Armitage test; not significant for tofacitinib, *p* = 0.00309 for abatacept. DAS28-ESR, Disease Activity Score in 28 joints using the erythrocyte sedimentation rate; SE 0, 0 copies of shared epitope alleles; SE 1, 1 copy of shared epitope alleles; SE 2, 2 copies of shared epitope alleles

**Fig. 5:**
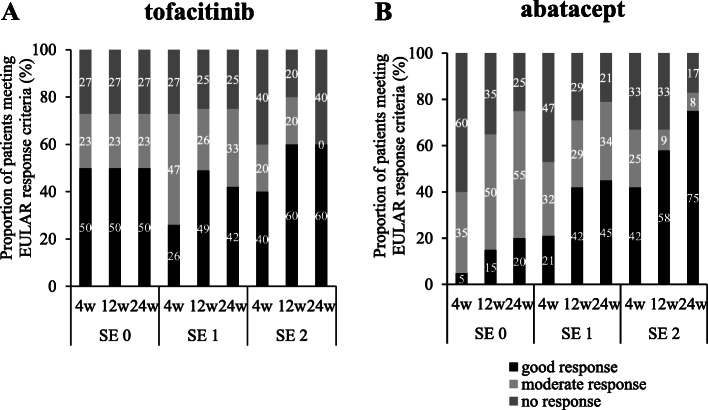
Copy numbers of SE alleles and EULAR response criteria. The percentage of patients meeting EULAR responses criteria stratified according to the copy numbers of SE alleles are shown 4, 12, and 24 weeks after the start of treatment with tofacitinib (**A**) or abatacept (**B**). The effects of the copy numbers of SE alleles on the percentage of patients who achieved a good EULAR response at 24 weeks in each treatment group was assessed by the Cochran-Armitage test; not significant for tofacitinib, *p* = 0.0182 for abatacept. EULAR, European League Against Rheumatism; SE 0, 0 copies of shared epitope alleles; SE 1, 1 copy of shared epitope alleles; SE 2, 2 copies of shared epitope alleles

Figure 4 shows the percentage of patients in DAS28-ESR remission with tofacitinib and abatacept stratified according to the copy numbers of SE alleles. The effects of the copy numbers of SE alleles on DAS28-ESR remission at week 24 were examined using the Cochran-Armitage test. The percentage of patients in DAS28-ESR remission at week 24 was not affected by the copy numbers of SE alleles in the tofacitinib group (32% for 0 SE alleles, 26% for 1 SE allele, and 40% for 2 SE alleles, *p* = 0.947), whereas it significantly increased as the copy numbers of SE alleles became higher in the abatacept group (10% for 0 SE alleles, 45% for 1 SE allele, and 58% for 2 SE alleles, *p* = 0.00309). These results were confirmed in an analysis using the EULAR response criteria shown in Fig. 5. The effects of the copy numbers of SE alleles on a good response versus a moderate or no response at week 24 were examined using the Cochran-Armitage test. The percentage of patients who achieved a good EULAR response at week 24 was not affected by the copy numbers of SE alleles in patients receiving tofacitinib (50% for 0 SE alleles, 42% for 1 SE allele, and 60% for 2 SE alleles, *p* = 0.924), but significantly increased as the copy numbers of SE alleles became higher in patients receiving abatacept (20% for 0 SE alleles, 45% for 1 SE allele, and 75% for 2 SE alleles, *p* = 0.0182).

### Impact of SE on DAS28-ESR remission

The impact of the presence of SE alleles on DAS28-ESR remission was assessed using a multivariable conditional logistic regression analysis adjusted for age, sex, RA disease duration, biologic-naïve, the ACPA titer, ΔDAS28-ESR, HAQ-DI, and PS. The presence of SE alleles (vs the absence of SE) correlated with achieving DAS28-ESR remission with abatacept at week 24 (OR = 25.881, 95% CI = 3.140–213.351, *p* = 0.0025), whereas the presence of SE did not affect responses to treatment with tofacitinib (OR = 1.473, 95% CI = 0.291–7.446, *p* = 0.639) (Table 2). Similarly, in the analysis using data before PS matching, the presence of SE alleles correlated with achieving DAS28-ESR remission with abatacept at week 24 (OR = 3.845, 95% CI = 1.386–10.669, *p* = 0.0097). In contrast, SE positivity did not affect responses to treatment with tofacitinib (OR = 1.910, 95% CI = 0.796–4.583, *p* = 0.1471) (see Supplementary Table S2).

**Table 2:** Impact of the presence of a shared epitope on DAS28-ESR remission at week 24 in a multivariable conditional logistic regression analysis

	OR	95% CI	*p* value
Tofacitinib	1.473	0.291–7.447	0.639
Abatacept	25.881	3.140–213.351	0.0025

The relationship between shared epitope (SE) positivity and DAS28-ESR remission was analyzed using a conditional logistic regression model adjusted for age, sex, RA disease duration, biologic-naïve, ACPA titer, ΔDAS28ESR HAQ-DI, and PS at baseline

*ACPA* anticitrullinated peptide antibody, ΔDAS28-ESR, delta DSA28-ESR, indicating the magnitude of changes from baseline to week 4 in DAS28-ESR; DAS28-ESR, Disease Activity Score in 28 joints using the erythrocyte sedimentation rate. *HAQ-DI* Health Assessment Questionnaires Disability Index, *PS* propensity score, *RF* rheumatoid factor, *SE* shared epitope

## Discussion

To the best of our knowledge, this is the first study to compare the clinical effectiveness of tofacitinib and abatacept. The clinically important results obtained demonstrated that tofacitinib and abatacept had similar clinical effectiveness at week 24, whereas tofacitinib exerted therapeutic effects earlier than abatacept. The significant clinical effects of tofacitinib assessed by ACR20 responses were previously detected as early as 2 weeks after the initiation of treatment [32]. Furthermore, a RCT comparing abatacept or infliximab with placebo reported that the onset of responses assessed by ACR20 initially appeared more rapidly with infliximab, whereas similar response rates were noted with abatacept and infliximab by day 85 [33]. Our results are consistent with these findings.

We also observed that the percentage of patients with EULAR good responses or DAS28-ESR remission significantly increased as the copy numbers of SE alleles became higher in patients receiving abatacept. In contrast, SE positivity had no effect on responses to treatment with tofacitinib. The different mechanistic roles of SE in response to these treatments may be attributed to differences in the mode of actions of both drugs. Previous studies reported that T cells were more strongly activated in ACPA-positive patients than in ACPA-negative patients [34], and SE alleles are more frequently detected in ACPA-positive patients (82–89.6%) than in ACPA-negative patients (53–70%) [35]. These findings suggest that T cells are activated more in SE-positive patients than in SE-negative patients. Therefore, abatacept, which selectively inhibits T cell activation, may be more effective in SE-positive patients. In contrast, tofacitinib has been shown to affect not only CD4^+^T cells, but also dendritic cells and B cells. Tofacitinib suppressed the production and stimulation of loop of a type-I interferon through JAK1/JAK3, decreased CD80/CD86 expression, and suppressed the T cell stimulatory capacity of dendritic cells. Furthermore, tofacitinib inhibited IgG production and IL-6 gene expression in the activated B cells [36]. Therefore, tofacitinib may exhibit its clinical effectiveness in SE-negative and -positive patients through pathways other than T cells.

Following the inclusion of SE positivity in a multivariable logistic analysis, the ACPA titter was not a significant predictor of DAS28-ESR remission at week 24 in patients receiving abatacept (see Supplementary Table S3). Since the presence of SE was significantly associated with ACPA titer (see Supplementary Table S6), we considered possible multicollinearity between ACPA and SE positivity and analyzed their impacts separately on achieving remission or low disease activity (LDA) at week 24 in patients receiving abatacept. We confirmed that the presence of SE significantly associated with the achievement of remission or LDA in DAS28-ESR, but ACPA positivity did not in conditional multivariable logistic analyses (see Supplementary Table S4 and S5). Previous studies reported a relationship between the increased efficacy of abatacept and ACPA positivity [37, 38]. Our results suggest that the better clinical response to abatacept in patients positive for ACPA observed in the previous studies may have been attributed to the presence of SE alleles, because the presence of SE were previously shown to be associated with the high titers of ACPA [9], which was also confirmed in the present study (see Supplementary Table S6). Since ACPA titers are also affected by non-SE alleles, such as HLA-DRB1 *0901 and *15 [39, 40], SE positivity may be a more accurate predictor of the efficacy of abatacept. In the present study, 30.0 and 25.7% of patients in the abatacept group had HLA-DR*0901 and *15, respectively.

Relationships have been reported between HLA-DRB1 alleles and clinical manifestations, including mortality risk [41], risk of severe joint destruction [10], and extra-articular manifestations, such as rheumatoid vasculitis [42]. Some amino acid haplotypes in HLA-DRB1 may be useful for the stratification of patients in terms of long-term outcomes, i.e., all-cause mortality, the risk of radiographic damage and laboratory measures of disease activity. Therefore, the prior identification of HLA-DRB1 alleles may be useful not only for selecting an appropriate molecular targeted therapy, but also for predicting disease progression in patients with RA in daily clinical practice. The present study has several limitations. First, we were unable to exclude all selection bias, even after PS matching. However, no significant differences were observed between the two treatment groups after PS matching. Furthermore, a certain number of cases were excluded after PS matching. We examined the relationship between SE and the effectiveness of these two drugs with and without PS matching and obtained similar results. In addition, due to the small sample size of this study, the caliper for PS matching was loosely set at width equal to 1 of the SD. However, a caliper width equal to 0.2–0.25 of the SD has generally been recommended [43, 44], and we performed PS matching again with a caliper width equal to 0.25 of the SD. After the PS matching, it was found that the impact of SE positivity on DAS28-ESR remission at week 24 in each treatment group was not different between patients selected with a caliper width equal to 0.25 of the SD and those selected with a caliper width equal to 1 of the SD. Second, all subjects in our study were Japanese descent and showed the similar proportion of patients with positive SE to the studies conducted in Korea [45], UK [46], and Sweden [47] except in a study conducted in Malaysia [48]. Proportion of RA patients with SE positivity may affect generalizability of our results in other regions or ethnicities. Third, the evaluation was set at week 24; however, longer observations may be required.

## Conclusions

The present study demonstrated that tofacitinib and abatacept had similar clinical effectiveness at week 24, whereas tofacitinib was superior to abatacept for changes from baseline and achieving DAS28-ESR remission at week 4. Furthermore, the presence of SE correlated with the achievement of DAS28-ESR remission at week 24 in patients receiving abatacept, but not in those receiving tofacitinib. Collectively, genetic information on HLA-DRB1 alleles and the present results are expected to be important factors facilitating shared decision making by rheumatologists discussing the advantages and disadvantages of different therapeutic options with their patients.

## Supplementary Information

**Additional file 1.** .

**Additional file 2.** .

## Data Availability

The datasets generated and/or analyzed in the present study are available from the corresponding author upon reasonable request.

## Abbreviations

ACPA: Anticitrullinated peptide antibody
ACR: American College of Rheumatology
CCP: Cyclic citrullinated peptide
CD: Cluster of differentiation
CDAI: Clinical Disease Activity Index
CRP: C-reactive protein
DAS28-ESR: Disease Activity Score using the erythrocyte sedimentation rate
DMARDs: Disease-modifying antirheumatic drugs
ESR: Erythrocyte sedimentation rate
EULAR: European League Against Rheumatism
HAQ-DI: Health Assessment Questionnaires Disability Index
HLA: Human leukocyte antigen
JAK: Janus kinase
RA: Rheumatoid arthritis
RF: Rheumatoid factor
SDAI: Simplified Disease Activity Index
SE: Shared epitope
