# Supplementary material for: Impact of the HLA-DRB1 shared epitope on responses to treatment with tofacitinib or abatacept in patients with rheumatoid arthritis
Source: Arthritis Res Ther. 2021 Aug 31;23:228. doi: 10.1186/s13075-021-02612-w (PMC8407060; doi:10.1186/s13075-021-02612-w)
Supplement: Supplementary file 1 — Additional file 1. . [file 13075_2021_2612_MOESM1_ESM.zip › ART_additional figure legend.docx]

**Supplementary figure legends**

**Supplementary Figure S1**

Drug retention rates of tofacitinib and abatacept. Retention rates for tofacitinib and abatacept over 24 weeks are shown.

**Supplementary Figure S2**

Time course of disease activity in CDAI and SDAI. The proportion of patients in the disease activity categories of CDAI (A) and SDAI (B) are shown at baseline, 4, 12, and 24 weeks after the initiation of treatment with tofacitinib or abatacept. CDAI, Clinical disease activity index; SDAI, Simplified Disease Activity Index.
