# Supplementary material for: Impact of the HLA-DRB1 shared epitope on responses to treatment with tofacitinib or abatacept in patients with rheumatoid arthritis
Source: Arthritis Res Ther. 2021 Aug 31;23:228. doi: 10.1186/s13075-021-02612-w (PMC8407060; doi:10.1186/s13075-021-02612-w)
Supplement: Supplementary file 1 — Additional file 1. . [file 13075_2021_2612_MOESM1_ESM.zip › ART_additonal figures.pptx]

## Slide 1
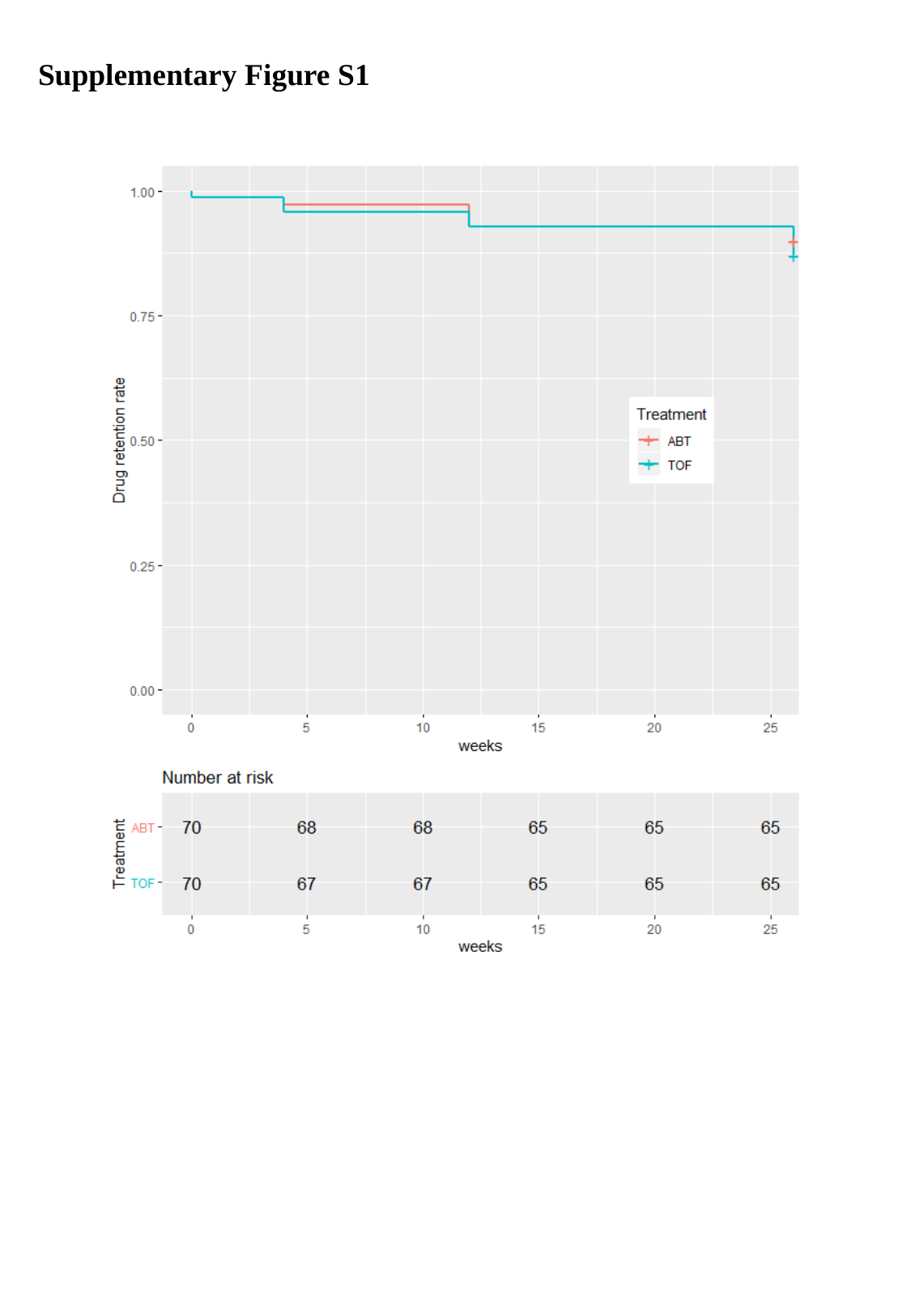

Supplementary Figure S1

## Slide 2
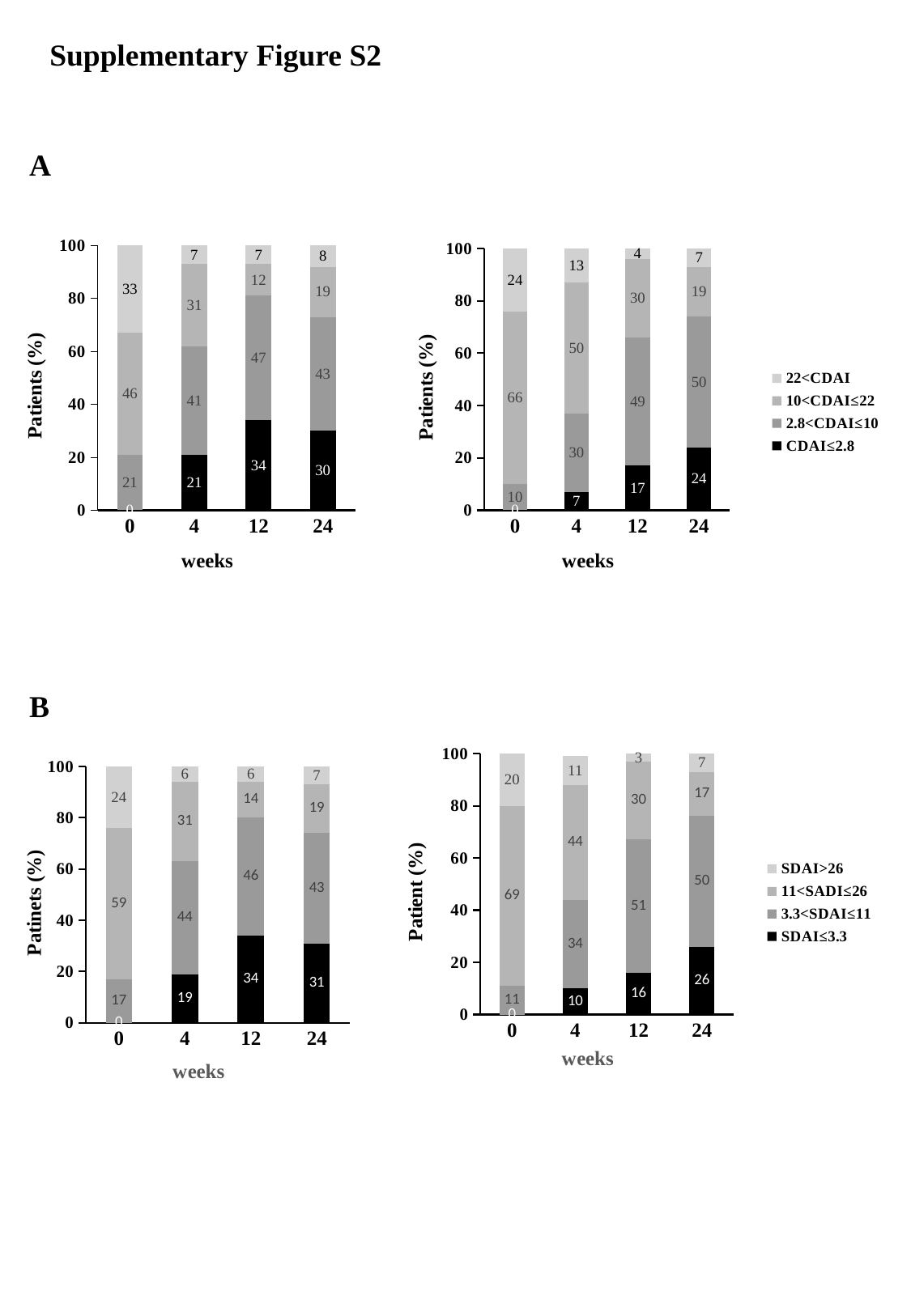

Supplementary Figure S2
A
### Chart
| Category | CDAI≤2.8 | 2.8<CDAI≤10 | 10<CDAI≤22 | 22<CDAI |
|---|---|---|---|---|
| 0 | 0.0 | 21.0 | 46.0 | 33.0 |
| 4 | 21.0 | 41.0 | 31.0 | 7.0 |
| 12 | 34.0 | 47.0 | 12.0 | 7.0 |
| 24 | 30.0 | 43.0 | 19.0 | 8.0 |
### Chart
| Category | CDAI≤2.8 | 2.8<CDAI≤10 | 10<CDAI≤22 | 22<CDAI |
|---|---|---|---|---|
| 0 | 0.0 | 10.0 | 66.0 | 24.0 |
| 4 | 7.0 | 30.0 | 50.0 | 13.0 |
| 12 | 17.0 | 49.0 | 30.0 | 4.0 |
| 24 | 24.0 | 50.0 | 19.0 | 7.0 |B
### Chart
| Category | SDAI≤3.3 | 3.3<SDAI≤11 | 11<SADI≤26 | SDAI>26 |
|---|---|---|---|---|
| 0 | 0.0 | 11.0 | 69.0 | 20.0 |
| 4 | 10.0 | 34.0 | 44.0 | 11.0 |
| 12 | 16.0 | 51.0 | 30.0 | 3.0 |
| 24 | 26.0 | 50.0 | 17.0 | 7.0 |
### Chart
| Category | SDAI≤3.3 | 3.3<SDAI≤11 | 11<SADI≤26 | SDAI>26 |
|---|---|---|---|---|
| 0 | 0.0 | 17.0 | 59.0 | 24.0 |
| 4 | 19.0 | 44.0 | 31.0 | 6.0 |
| 12 | 34.0 | 46.0 | 14.0 | 6.0 |
| 24 | 31.0 | 43.0 | 19.0 | 7.0 |
