# Supplementary material for: Impact of the HLA-DRB1 shared epitope on responses to treatment with tofacitinib or abatacept in patients with rheumatoid arthritis
Source: Arthritis Res Ther. 2021 Aug 31;23:228. doi: 10.1186/s13075-021-02612-w (PMC8407060; doi:10.1186/s13075-021-02612-w)
Supplement: Supplementary file 1 — Additional file 1. . [file 13075_2021_2612_MOESM1_ESM.zip › ART_revised Figure.pptx]

## Slide 1
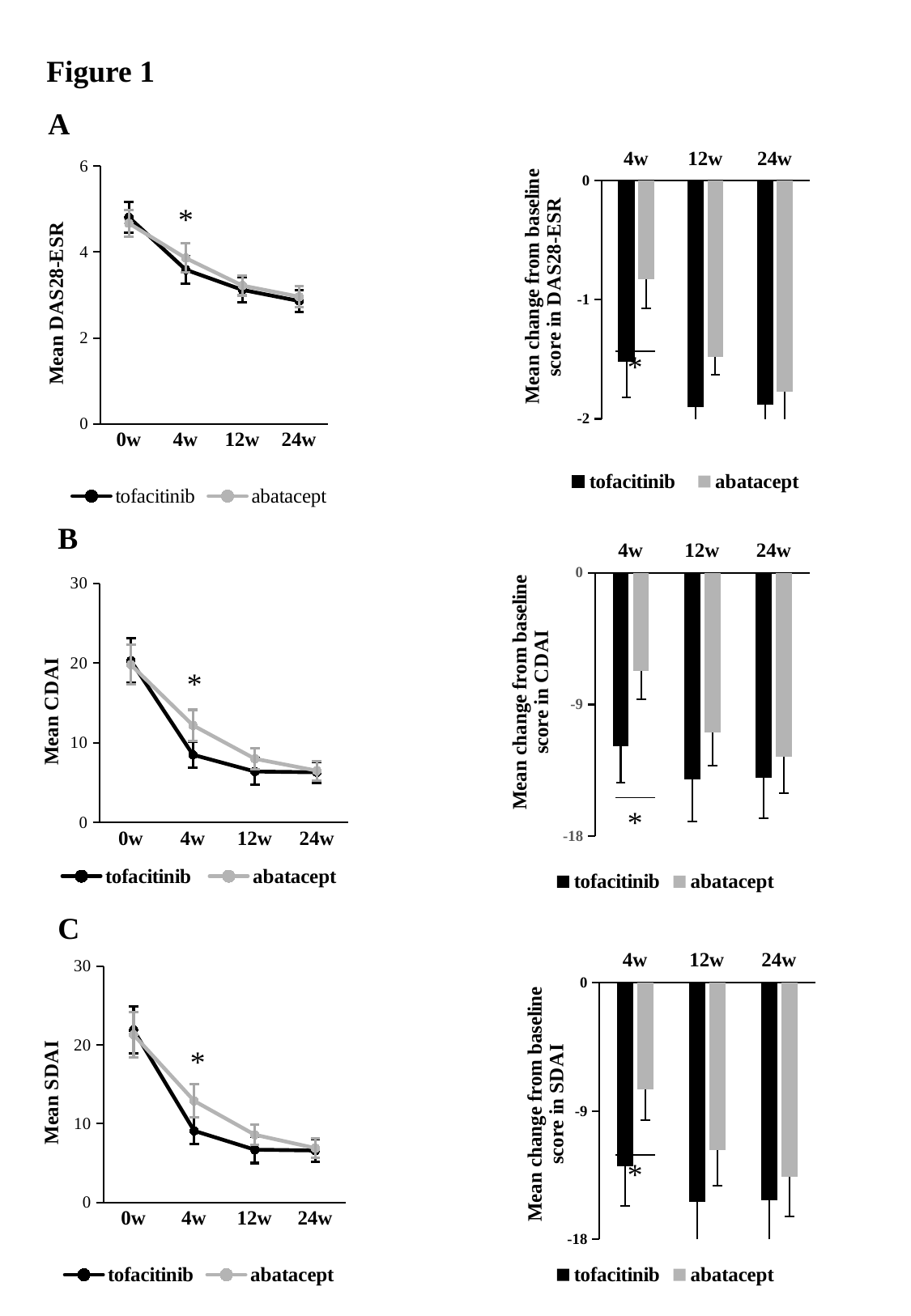

Figure 1
A
### Chart
| Category | tofacitinib | abatacept |
|---|---|---|
| 0w | 4.81 | 4.67 |
| 4w | 3.59 | 3.86 |
| 12w | 3.12 | 3.22 |
| 24w | 2.86 | 2.96 |
### Chart
| Category | tofacitinib | abatacept |
|---|---|---|
| 4w | -1.52 | -0.83 |
| 12w | -1.9 | -1.48 |
| 24w | -1.88 | -1.77 | *
*
B
### Chart
| Category | tofacitinib | abatacept |
|---|---|---|
| 4w | -11.86 | -6.73 |
| 12w | -14.1 | -10.9 |
| 24w | -13.99 | -12.56 |
### Chart
| Category | tofacitinib | abatacept |
|---|---|---|
| 0w | 20.3 | 19.8 |
| 4w | 8.5 | 12.2 |
| 12w | 6.4 | 8.0 |
| 24w | 6.3 | 6.5 |*
*
C
### Chart
| Category | tofacitinib | abatacept |
|---|---|---|
| 4w | -12.86 | -7.46 |
| 12w | -15.39 | -11.75 |
| 24w | -15.25 | -13.61 |
### Chart
| Category | tofacitinib | abatacept |
|---|---|---|
| 0w | 21.9 | 21.3 |
| 4w | 9.1 | 12.9 |
| 12w | 6.7 | 8.6 |
| 24w | 6.6 | 6.9 |*
*

## Slide 2
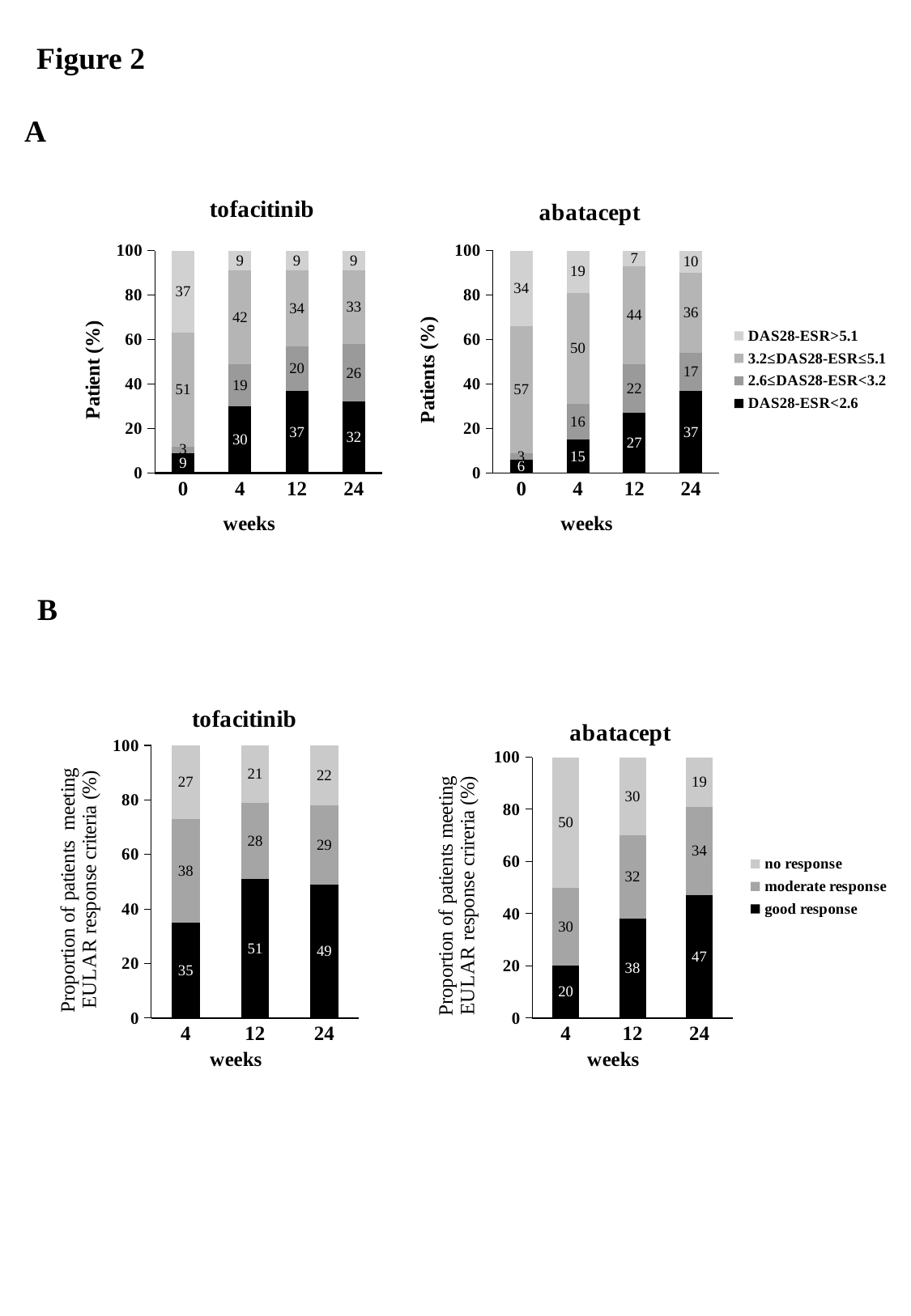

Figure 2
A
### Chart: tofacitinib
| Category | DAS28ESR<2.6 | 2.6≤DAS28ESR<3.2 | 3.2≤DAS28ESR≤5.1 | DAS28ESR>5.1 |
|---|---|---|---|---|
| 0 | 9.0 | 3.0 | 51.0 | 37.0 |
| 4 | 30.0 | 19.0 | 42.0 | 9.0 |
| 12 | 37.0 | 20.0 | 34.0 | 9.0 |
| 24 | 32.0 | 26.0 | 33.0 | 9.0 |
### Chart: abatacept
| Category | DAS28-ESR<2.6 | 2.6≤DAS28-ESR<3.2 | 3.2≤DAS28-ESR≤5.1 | DAS28-ESR>5.1 |
|---|---|---|---|---|
| 0 | 6.0 | 3.0 | 57.0 | 34.0 |
| 4 | 15.0 | 16.0 | 50.0 | 19.0 |
| 12 | 27.0 | 22.0 | 44.0 | 7.0 |
| 24 | 37.0 | 17.0 | 36.0 | 10.0 |B
### Chart: tofacitinib
| Category | good | moderate | no |
|---|---|---|---|
| 4 | 35.0 | 38.0 | 27.0 |
| 12 | 51.0 | 28.0 | 21.0 |
| 24 | 49.0 | 29.0 | 22.0 |
### Chart: abatacept
| Category | good response | moderate response | no response |
|---|---|---|---|
| 4 | 20.0 | 30.0 | 50.0 |
| 12 | 38.0 | 32.0 | 30.0 |
| 24 | 47.0 | 34.0 | 19.0 |

## Slide 3
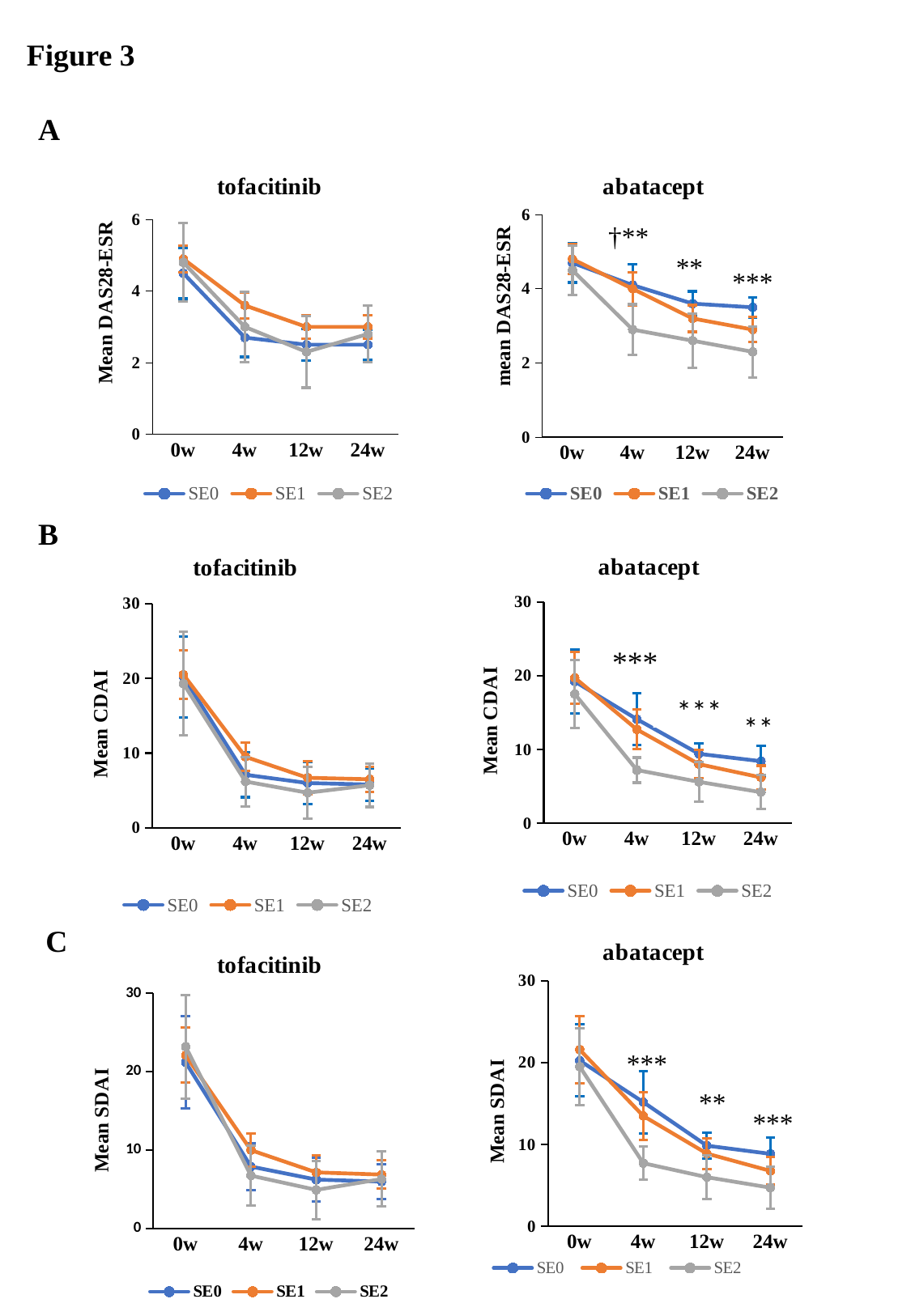

Figure 3
A
### Chart: abatacept
| Category | SE0 | SE1 | SE2 |
|---|---|---|---|
| 0w | 4.7 | 4.8 | 4.5 |
| 4w | 4.1 | 4.0 | 2.9 |
| 12w | 3.6 | 3.2 | 2.6 |
| 24w | 3.5 | 2.9 | 2.3 |
### Chart: tofacitinib
| Category | SE0 | SE1 | SE2 |
|---|---|---|---|
| 0w | 4.5 | 4.9 | 4.8 |
| 4w | 2.7 | 3.6 | 3.0 |
| 12w | 2.5 | 3.0 | 2.3 |
| 24w | 2.5 | 3.0 | 2.8 | †**
 **
***
B
### Chart: abatacept
| Category | SE0 | SE1 | SE2 |
|---|---|---|---|
| 0w | 19.2 | 19.7 | 17.5 |
| 4w | 14.1 | 12.7 | 7.2 |
| 12w | 9.4 | 8.0 | 5.6 |
| 24w | 8.4 | 6.2 | 4.2 |
### Chart: tofacitinib
| Category | SE0 | SE1 | SE2 |
|---|---|---|---|
| 0w | 20.2 | 20.5 | 19.3 |
| 4w | 7.1 | 9.5 | 6.2 |
| 12w | 6.0 | 6.7 | 4.7 |
| 24w | 5.8 | 6.5 | 5.7 |***
***
***
 ***
**
 **
C
### Chart: abatacept
| Category | SE0 | SE1 | SE2 |
|---|---|---|---|
| 0w | 20.238 | 21.57 | 19.5 |
| 4w | 15.149 | 13.453 | 7.691 |
| 12w | 9.827 | 8.863 | 5.978 |
| 24w | 8.829 | 6.744 | 4.692 |
### Chart: tofacitinib
| Category | SE0 | SE1 | SE2 |
|---|---|---|---|
| 0w | 21.214 | 22.11 | 23.2 |
| 4w | 7.92 | 10.008 | 6.76 |
| 12w | 6.253 | 7.175 | 4.942 |
| 24w | 6.005 | 6.895 | 6.322 |　***
 　**
　***

## Slide 4
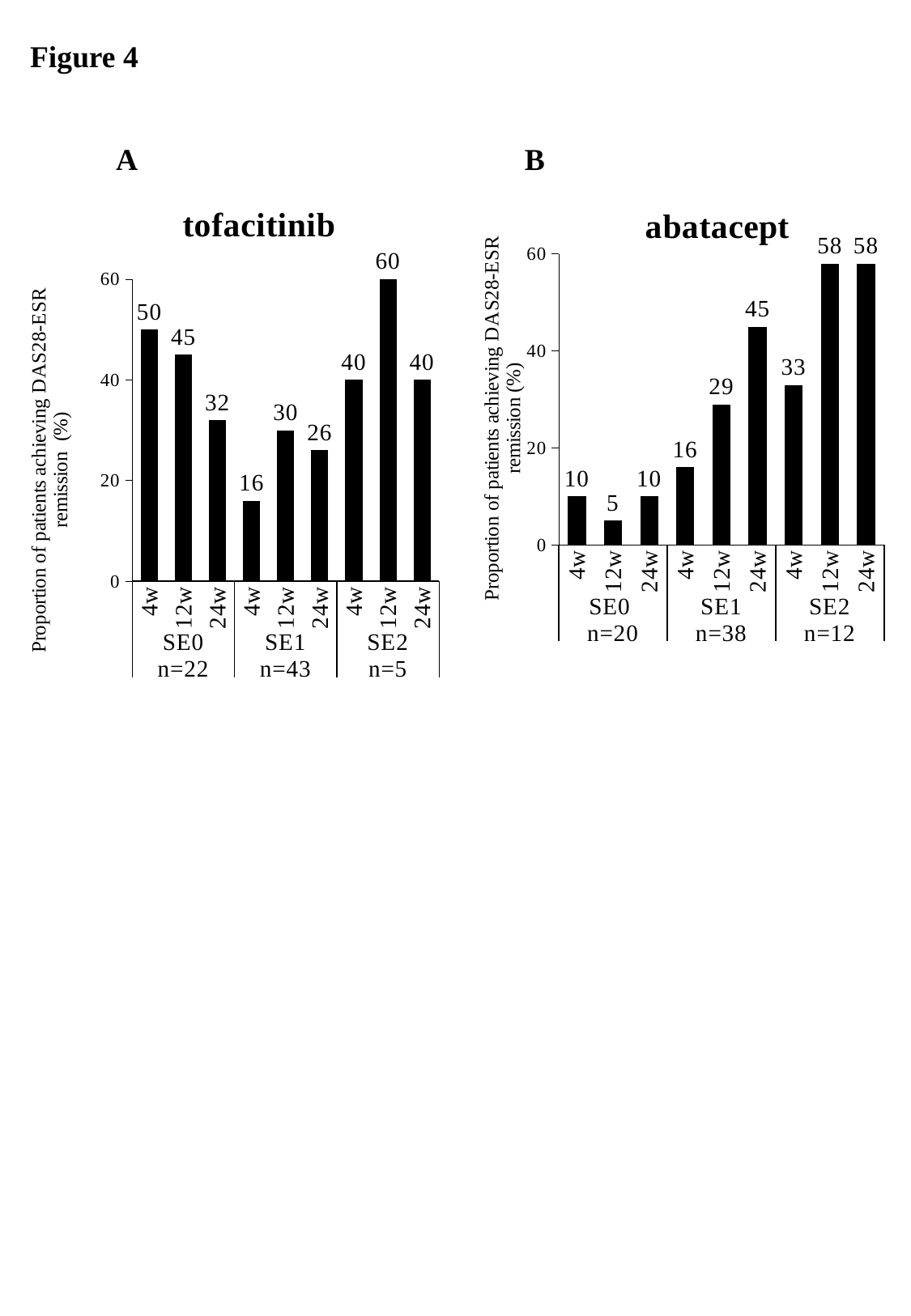

Figure 4
B
A
### Chart: tofacitinib
| Category | TOF |
|---|---|
| 4w | 50.0 |
| 12w | 45.0 |
| 24w | 32.0 |
| 4w | 16.0 |
| 12w | 30.0 |
| 24w | 26.0 |
| 4w | 40.0 |
| 12w | 60.0 |
| 24w | 40.0 |
### Chart: abatacept
| Category | ABT |
|---|---|
| 4w | 10.0 |
| 12w | 5.0 |
| 24w | 10.0 |
| 4w | 16.0 |
| 12w | 29.0 |
| 24w | 45.0 |
| 4w | 33.0 |
| 12w | 58.0 |
| 24w | 58.0 |

## Slide 5
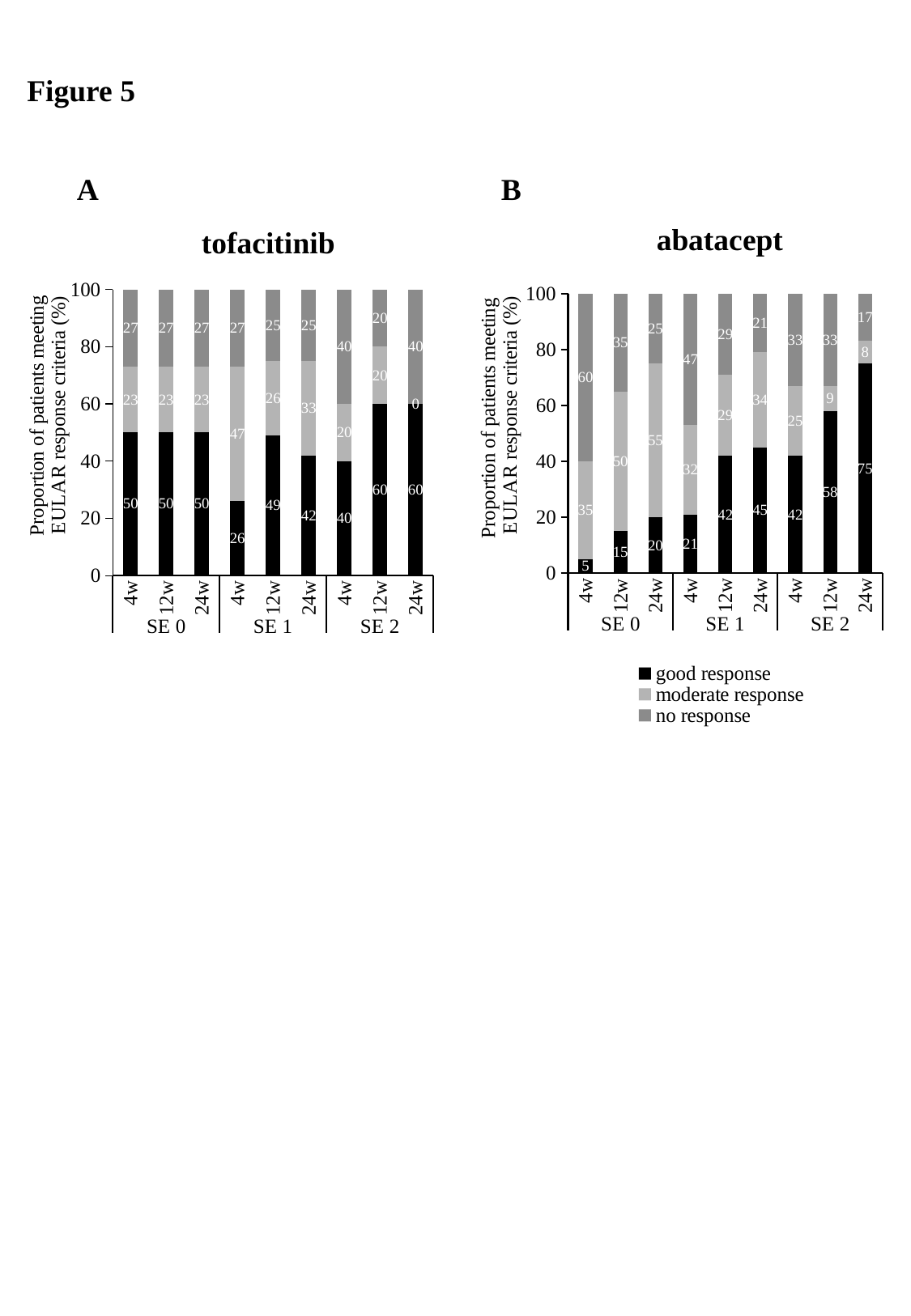

Figure 5
B
A
abatacept
tofacitinib
### Chart
| Category | good response | moderate response | no response |
|---|---|---|---|
| 4w | 5.0 | 35.0 | 60.0 |
| 12w | 15.0 | 50.0 | 35.0 |
| 24w | 20.0 | 55.0 | 25.0 |
| 4w | 21.0 | 32.0 | 47.0 |
| 12w | 42.0 | 29.0 | 29.0 |
| 24w | 45.0 | 34.0 | 21.0 |
| 4w | 42.0 | 25.0 | 33.0 |
| 12w | 58.0 | 9.0 | 33.0 |
| 24w | 75.0 | 8.0 | 17.0 |
### Chart
| Category | good | moderate | no |
|---|---|---|---|
| 4w | 50.0 | 23.0 | 27.0 |
| 12w | 50.0 | 23.0 | 27.0 |
| 24w | 50.0 | 23.0 | 27.0 |
| 4w | 26.0 | 47.0 | 27.0 |
| 12w | 49.0 | 26.0 | 25.0 |
| 24w | 42.0 | 33.0 | 25.0 |
| 4w | 40.0 | 20.0 | 40.0 |
| 12w | 60.0 | 20.0 | 20.0 |
| 24w | 60.0 | 0.0 | 40.0 |
