# Supplementary material for: Impact of the HLA-DRB1 shared epitope on responses to treatment with tofacitinib or abatacept in patients with rheumatoid arthritis
Source: Arthritis Res Ther. 2021 Aug 31;23:228. doi: 10.1186/s13075-021-02612-w (PMC8407060; doi:10.1186/s13075-021-02612-w)
Supplement: Supplementary file 2 — Additional file 2. . [file 13075_2021_2612_MOESM2_ESM.docx]

**Supplementary Table S1**

Baseline characteristics of patients before propensity score matching

| Variables | tofacitinib (n=164) | abatacept (n=131) | *P* |
| --- | --- | --- | --- |
| Age, years | 66.0±11.5 | 71.1±11.1 | 1.55x10^-4^ |
| Female, n (%) | 141 (86.0) | 110 (84.0) | 0.743 |
| Disease duration, years | 14.7±11.7 | 14.7±14.0 | 0.311 |
| Stage I/II/III/IV, % | 18.9/7.3/37.8/36.0 | 19.8/15.3/31.3/33.6 | 0.155 |
| Class 1/2/3/4, % | 4.3/76.2/19.5/0.0 | 6.9/68.0/24.4/0.0 | 0.305 |
| BMI, kg/m² | 22.1±3.9 | 21.4±3.0 | 0.202 |
| SE copy number 0/1/2, % | 25.6/61.6/12.8 | 35.1/49.6/15.3 | 0.113 |
| Current smoker, n (%) | 10 (6.1) | 8 (6.1) | 1.00 |
| Ever smoker, n (%) | 42 (25.6) | 35 (26.7) | 0.894 |
| No. of prior biologic use  0 (biologic naïve)  1  2  ≥3 | 32  54  36  42 | 96  15  8  12 | 2.08x10^-20^  1.49x10^-15^  0.000147  0.000273 |
| MTX use, n (%) | 100 (61.0) | 74 (56.5) | 0.476 |
| MTX dose, mg/week | 8.4±2.5 | 8.0±2.4 | 0.284 |
| Oral steroid use, n (%) | 63 (26.2) | 44 (33.6) | 0.446 |
| Oral steroid dose, mg/day | 4.3±2.3 | 4.8±3.1 | 0.272 |
| MMP-3, ng/mL | 206.0±216.2 | 200.4±223.7 | 0.733 |
| SJC, 0–28 | 3.9±4.0 | 4.5±3.9 | 0.632 |
| TJC, 0–28 | 5.6±4.9 | 5.6±5.0 | 0.970 |
| ESR, mm/h | 37.5±30.6 | 41.8±29.0 | 0.0886 |
| CRP, mg/dL | 1.63±2.18 | 1.89±2,52 | 0.0419 |
| RF, U/mL | 191.0±394.1 | 171.8±307.2 | 0.541 |
| ACPA, U/mL | 218.7±304.6 | 318.2±388.9 | 0.0419 |
| GH, VAS 0–100 mm | 55.1±27.3 | 53.2±23.5 | 0.441 |
| EGA, VAS 0–100 mm | 50.0±21.3 | 48.8±16.8 | 0.0476 |
| SDAI | 21.8±12.1 | 21.7±11.3 | 0.992 |
| CDAI | 20.2±11.0 | 19.8±9.7 | 0.935 |
| DAS28 ESR | 4.7±1.5 | 4.9±1.2 | 0.240 |
| HAQ-DI | 1.0±0.7 | 1.2±0.8 | 0.622 |

*Prednisolone equivalents.

Results are expressed as means±SD unless otherwise stated.

ACPA, anticitrullinated peptide antibody; BMI, body mass index; CRP, C-reactive protein; CDAI, Clinical Disease Activity Index; DAS28-ESR,

Disease Activity Score in 28 joints using the erythrocyte sedimentation rate; EGA, evaluator global assessment of disease activity; ESR, erythrocyte sedimentation rate; GH, patient’s global assessment of general health; HAQ-DI, Health Assessment Questionnaire Disability Index; MMP-3, matrix metalloproteinase 3; MTX, methotrexate; RF, rheumatoid factor; SE, shared epitope; SDAI, Simplified Disease Activity Index; SJC, swollen joint count; TJC, tender joint count.

**Supplementary Table S2**

**Impact of the presence of a shared epitope on DAS28-ESR remission at week 24 in a multivariable conditional logistic analysis before propensity-score matching**

|  | OR | 95%CI | *P*-value |
| --- | --- | --- | --- |
| Tofacitinib | 1.910 | 0.796−4.582 | 0.1471 |
| Abatacept | 3.845 | 1.386−10.669 | 0.0097 |

The relationship between shared epitope (SE) positivity and DAS28-ESR remission was analyzed using a conditional logistic regression model adjusted for age, sex, RA disease duration, biologic-naïve, the ACPA titer, ΔDAS28ESR, HAQ-DI and PS at baseline.

ACPA, anticitrullinated peptide antibody, ΔDAS28-ESR, delta DSA28-ESR, indicating the magnitude of changes from baseline to week 4 in DAS28-ESR; DAS28-ESR, Disease Activity Score in 28 joints using the erythrocyte sedimentation rate. HAQ-DI, Health Assessment Questionnaires Disability Index; PS, propensity score; RF, rheumatoid factor; SE, shared epitope.

**Supplementary Table S3**

**Predictors for DAS28-ESR remission in patients treated with abatacept by a multivariable conditional logistic analysis**

| Variables | OR | 95%CI | *p*-value |
| --- | --- | --- | --- |
| Shared epitope positivity | 25.881 | 3.140−213.351 | 0.0025 |
| ACPA titer | 1.022 | 1.000−1.005 | 0.0583 |
| Age | 0.928 | 0.865−0.995 | 0.0354 |
| Sex | 1.200 | 0.194−7.439 | 0.845 |
| RA disease duration | 0.986 | 0.929−1.046 | 0.637 |
| Biologic-naïve | 0.986 | 0.172−5.650 | 0.988 |
| HAQ-DI | 0.398 | 0.134−1.178 | 0.0962 |
| ΔDAS28-ESR | 0.697 | 0.342−1.419 | 0.319 |

Predictive factors for DAS28-ESR remission at week 24 in patients treated with abatacept were examined using a conditional logistic regression model adjusted for age, sex, RA disease duration, biologic-naïve, shared epitope positivity, the ACPA titer, ΔDAS28-ESR, HAQ-DI and PS at baseline.

ACPA, anticitrullinated peptide antibody; ΔDAS28-ESR, delta DAS28-ESR, indicating the magnitude of changes from baseline to week 4 in DAS28-ESR; DAS28-ESR, Disease Activity Score in 28 joints using erythrocyte sedimentation rate; HAQ-DI, Health Assessment Questionnaires Disability Index; PS, propensity score; RA, rheumatoid arthritis.

**Supplementary Table S4**

**Impact of ACPA on DAS28-ESR remission or LDA in patients receiving abatacept by a multivariable conditional logistic analysis**

| Variables | OR | 95%CI | *p*-value |
| --- | --- | --- | --- |
| ACPA positivity | 5.170 | 0.895−29.877 | 0.0664 |
| Age | 0.970 | 0.917−1.027 | 0.3011 |
| Sex | 1.633 | 0.372−7.169 | 0.5159 |
| RA disease duration | 1.008 | 0.960−1.058 | 0.7558 |
| Biologic naïve | 3.090 | 0.372−25.647 | 0.2960 |
| ΔDAS28-ESR | 0.782 | 0.425−1.440 | 0.4298 |
| HAQ-DI | 0.596 | 0.252−1.411 | 0.2396 |

Predictive factors for remission or LDA in DAS28-ESR at week 24 in patients receiving abatacept were examined using a conditional logistic regression model adjusted for age, sex, RA disease duration, biologic naïve, ACPA positivity, ΔDAS28-ESR, HAQ-DI and PS at baseline. ACPA, anticitrullinated peptide antibody; ΔDAS28-ESR, delta DAS28-ESR, indicating the magnitude of changes from baseline to week 4 in DAS28-ESR; DAS28-ESR, Disease Activity Score in 28 joints using erythrocyte sedimentation rate; LDA, Low disease activity; HAQ-DI, Health Assessment Questionnaires Disability Index; PS, propensity score; RA, rheumatoid arthritis.

**Supplementary Table S5**

**Impact of shared epitope on DAS 28-ESR remission or LDA in patients receiving abatacept by a multivariable conditional logistic analysis**

| Variables | OR | 95%CI | *p*-value |
| --- | --- | --- | --- |
| Shared epitope positivity | 8.986 | 1.999−40.390 | 0.0042 |
| Age | 0.966 | 0.911−1.024 | 0.2450 |
| Sex | 3.005 | 0.600−15.039 | 0.1806 |
| RA disease duration | 0.996 | 0.943−1.052 | 0.8839 |
| Biologic naïve | 1.171 | 0.121−11.327 | 0.8914 |
| ΔDAS28-ESR | 0.814 | 0.424−1.564 | 0.5374 |
| HAQ-DI | 0.362 | 0.136−0.963 | 0.0417 |

Predictive factors for remission or LDA in DAS28-ESR at week 24 in patients receiving abatacept were examined using a conditional logistic regression model adjusted for age, sex, RA disease duration, biologic naïve, shared epitope positivity; ΔDAS28-ESR, HAQ-DI and PS at baseline. ΔDAS28-ESR, delta DAS28-ESR, indicating the magnitude of changes from baseline to week 4 in DAS28-ESR; DAS28-ESR, Disease Activity Score in 28 joints using erythrocyte sedimentation rate; LDA, Low disease activity; HAQ-DI, Health Assessment Questionnaires Disability Index; PS, propensity score; RA, rheumatoid arthritis.

**Supplementary Table S6**

**Baseline characteristics of patients before propensity score matching stratified by copy numbers of SE alleles**

| Variables | Copy numbers of SE alleles | | | *P* value¹ |
| --- | --- | --- | --- | --- |
|  | 0 copies (n=88) | 1 copy (n=166) | 2 copies (n=41) |  |
| Age, years | 68.3±12.4 | 68.5±11.2 | 67.5±12,1 | 0.921 |
| Female, n (%) | 79 (90.0) | 135 (81.3) | 37 (90.2) | 0.121 |
| Disease duration, years | 14.2±13.3 | 14.8±12.2 | 15.3±13.8 | 0.728 |
| Stage I/II/III/IV, % | 25.0/6.8/36.4/31.8 | 16.9/13.3/34.3/35.5 | 17.1/9.8/34.1/39.0 | 0.560 |
| Class 1/2/3/4, % | 5.7/78.4/15.9/0.0 | 4.2/72.1/23.6/0.0 | 9.8/63.4/26.8/0.0 | 0.404 |
| BMI, kg/m² | 22.2±3.7 | 21.7±3.5 | 21.5±3.5 | 0.220 |
| Current smoker, n (%) | 6 (6.8) | 12 (7.2) | 0 (0.0) | 0.211 |
| Ever smoker, n (%) | 22 (25.0) | 45 (27.1) | 10 (24.4) | 0.903 |
| Number of failed bDMARD | 1.13±1.33 | 1.24±1.39 | 1.02±1.41 | 0.502 |
| MTX use, n (%) | 55 (62.5) | 90 (54.2) | 29 (70.7) | 0.114 |
| MTX dose, mg/week | 7.9±2.3 | 8.5±2.4 | 7.7±2.8 | 0.212 |
| Oral steroid use, n (%) | 33 (37.5) | 57 (34.3) | 16 (39.0) | 0.800 |
| Oral steroid dose, mg/day* | 4.3±1.8 | 4.7±3.2 | 4.2±2.0 | 0.873 |
| MMP-3, ng/mL | 192.8±213.7 | 210.5±230.3 | 199.1±218.4 | 0.854 |
| SJC, 0−28 | 4.3±3.6 | 4.0±4.0 | 4.6±4.5 | 0.499 |
| TJC, 0−28 | 6.3±5.7 | 5.4±4.6 | 5.0±4.8 | 0.505 |
| ESR, mm/h | 34.8±26.4 | 41.9±30.6 | 39.3±33.5 | 0.237 |
| CRP, mg/dL | 1.32±1.83 | 1.94±2.46 | 1.85±2.71 | 0.130 |
| RF, U/mL | 162.2±266.3 | 180.8±318.5 | 232.6±603.5 | 0.189 |
| ACPA, U/mL | 174.1±287.3 | 293.3±371.8 | 330.5±334.5 | <0.0011 |
| GH, VAS 0−100 mm | 53.0±26.9 | 54.9±24.8 | 54.5±29.2 | 0.825 |
| EGA, VAS 0−100 mm | 45.8±18.8 | 48.6±19.1 | 48.0±23.2 | 0.598 |
| SDAI | 21.8±11.0 | 21.8±11.8 | 21.7±13.0 | 0.990 |
| CDAI | 20.5±10.1 | 19.8±10.4 | 19.9±11.3 | 0.809 |
| DAS28 ESR | 4.7±1.4 | 4.8±1.3 | 4.6±1.5 | 0.710 |
| HAQ-DI | 0.95±0.74 | 1.10±0.78 | 1.06±0.83 | 0.268 |

*Prednisolone equivalents.

Results are expressed as means±SD unless otherwise stated.

^1^Differences in the distribution of the three groups stratified according to the copy numbers of SE alleles were examined by the Kruskal-Wallis test.

ACPA, anticitrullinated peptide antibody; bDMARD, biologic disease modifying antirheumatic drug; BMI, body mass index; CRP, C-reactive protein; CDAI, Clinical Disease Activity Index; DAS28-ESR, Disease Activity Score in 28 joints using the erythrocyte sedimentation rate; EGA, evaluator global assessment of disease activity; ESR, erythrocyte sedimentation rate; GH, patient’s global assessment of general health; HAQ-DI, Health Assessment Questionnaire Disability Index; MMP-3, matrix metalloproteinase 3; MTX, methotrexate; RF, rheumatoid factor; SE, shared epitope; SDAI, Simplified Disease Activity Index; SJC, swollen joint count; TJC, tender joint count.
